# *Andrographis paniculata* and Its Bioactive Diterpenoids Against Inflammation and Oxidative Stress in Keratinocytes

**DOI:** 10.3390/antiox9060530

**Published:** 2020-06-17

**Authors:** Eugenie Mussard, Sundy Jousselin, Annabelle Cesaro, Brigitte Legrain, Eric Lespessailles, Eric Esteve, Sabine Berteina-Raboin, Hechmi Toumi

**Affiliations:** 1 Laboratory I3MTO, EA 4708, Université d’Orléans, CEDEX 2, 45067 Orléans, France; eugenie.mussard@univ-orleans.fr (E.M.); sundy.jousselin@univ-orleans.fr (S.J.); annabelle.cesaro@univ-orleans.fr (A.C.); eric.lespessailles@chr-orleans.fr (E.L.); 2 NOVAXIA-6 Rue des Champs Godin, 41220 St Laurent Nouan, France; b.legrain@labo-novaxia.com; 3 Service de Rhumatologie, Centre Hospitalier Régional d’Orléans CHRO, 14 Avenue de l’Hôpital, 45100 Orléans, France; 4 Plateforme Recherche Innovation Médicale Mutualisée d’Orléans, Centre Hospitalier Régional d’Orléans 14 Avenue de l’Hôpital, 45100 Orléans, France; 5 Service de Dermatologie, Centre Hospitalier Régional d′Orléans 14 Avenue de l’Hôpital, 45100 Orléans, France; eric.esteve@chr-orleans.fr; 6 Institut de Chimie Organique et Analytique ICOA, Université d’Orléans-Pôle de Chimie, UMR CNRS 7311, Rue de Chartres-BP 6759, CEDEX 2, 45067 Orléans, France; sabine.berteina-raboin@univ-orleans.fr

**Keywords:** *Andrographis paniculata*, andrographolide, skin aging, keratinocytes, oxidative stress, inflammation

## Abstract

*Andrographis paniculata* was widely used in traditional herbal medicine to treat various diseases. This study explored the potential anti-aging activity of *Andrographis paniculata* in cutaneous cells. Human, adult, low calcium, high temperature (HaCaT) cells were treated with methanolic extract (ME), andrographolide (ANDRO), neoandrographolide (NEO), 14-deoxyandrographolide (14DAP) and 14-deoxy-11,12-didehydroandrographolide (14DAP11-12). Oxidative stress and inflammation were induced by hydrogen peroxide and lipopolysaccharide/TNF-α, respectively. Reactive oxygen species (ROS) production was measured by fluorescence using a 2′,7′-dichlorofluorescein diacetate (DCFH-DA) probe and cytokines were quantified by ELISA for interleukin-8 (IL-8) or reverse transcription-quantitative polymerase chain reaction (RT-qPCR) for tumor necrosis factor-α (TNF-α). Hyaluronic acid (HA) secretion was determined by an ELISA. Our results show a decrease in ROS production and TNF-α expression by ME (5 µg/mL) in HaCaT under pro-oxidant and pro-inflammatory conditions, respectively. ME protected HaCaT against oxidative stress and inflammation. Our findings confirm that ME can be used for the development of bioactive compounds against epidermal damage.

## 1. Introduction

The skin is the largest human organ, the interface between the body and the environment. With age, the skin suffers natural wear and tear, just like the rest of our body. Skin aging has both intrinsic and extrinsic origins [1,2]. The former correspond to the fiber meshwork of collagen and elastin (intrinsic aging) and the latter to the environmental factors that age our skin such as ultraviolet light. In both processes, oxidative stress plays a role in inflammation induction and the destruction of skin proteins [3,4]. This results in a decrease in the renewal of epidermal cells and consequently a decrease in epidermal thickness [5]. Keratinocytes are the main cells of the epidermis. Extrinsic factors, such as solar radiation, induce the production of reactive oxygen species (ROS). ROS affect keratinocytes, which in turn secrete pro-inflammatory cytokines and produce more ROS [6]. Inflammation and oxidative stress also cause the production and activation of matrix metalloproteinases (MMPs). MMPs damage the extracellular matrix (ECM) in the dermis [3]. Some of the cytokines known to be released by keratinocytes when the skin is in inflammatory condition are interleukin-8 (IL-8) and tumor necrosis factor-α (TNF-α) [7,8]. Hyaluronic acid (HA) is one of the main components of the extracellular matrix of the skin. It is present in the dermis but also in the epidermis. With age, epidermis degradation leads to skin dehydration [9].

*Andrographis paniculata* (Burm. F.), commonly called “king of bitters”, is a plant of the Acanthaceae family. This plant has been used in traditional medicine in Asia to treat inflammatory diseases such as respiratory disorders [10]. Methanolic extract (ME) from the leaves of *Andrographis paniculata* has been shown to have antioxidant and anti-inflammatory activities [11,12,13,14]. Andrographolide (ANDRO) is the main bioactive compound of *Andrographis paniculata* [15]. In the context of skin aging, ANDRO is interesting for its antioxidant and anti-inflammatory activities [14,16,17,18]. ANDRO is a diterpenoid, like neoandrographolide (NEO), 14-deoxyandrographolide (14DAP), and 14-deoxy-11,12-didehydroandrographolide (14DAP11-12). These compounds have antioxidant and anti-inflammatory properties [19,20,21,22,23]. 

ME and 14DAP have been shown to decrease ROS production under oxidative stress conditions, as well as IL-6 secretion and TNF-α expression under inflammatory conditions, in dermal fibroblasts [14]. However, there is poor information on epidermal cells and keratinocytes. In this study, our objective was to evaluate the anti-aging properties of ME, ANDRO, NEO, 14DAP, and 14DAP11-12 (Table 1) on HaCaT.

## 2. Materials and Methods 

### 2.1. Chemicals

Andrographolide was obtained from Sigma-Aldrich (Sigma-Aldrich, Saint-Louis, MO, USA). Neoandrographolide, 14-deoxyandrographolide and 14-deoxy-11,12-didehydroandrographolide were purchased from Carbosynth (Carbosynth, Compton, Berkshire, UK). The molecules were dissolved in dimethyl sulfoxide (DMSO) as a stock solution at 10 mg/mL and stored at −20 °C. 

### 2.2. Preparation of Andrographis paniculata Extract

Dried leaves of *Andrographis paniculata* were purchased from AYur-vana®. The leaf powder was extracted by maceration in methanol (15 mL/g) for 2h at RT (room temperature) and then sonicated for 1h in ice. The crude extract was filtered, and the methanol was evaporated overnight at RT. The extract was suspended in DMSO (0.6 g/mL) and filtered. 

### 2.3. HPLC Analysis

ME was analyzed by reverse-phase HPLC (High Performance Liquid Chromatography) using a Zorbax Eclipse XDB-C18 column 4.6 × 150 mm (Agilent) on an Agilent 1220 Infinity II LC System. The mobile phase was delivered at a rate of 1 mL/min with a gradient from A (0.1% HCOOH in H_2_O) to B (0.1% HCOOH in CH_3_CN) (10% B for 4 min, 10% to 60% B in 10 min, 60% to 100% B in 2 min). The column effluent was monitored at 250 nm.

The quantity of ANDRO contained in the ME was determined by comparison with a range of pure ANDRO (standard).

### 2.4. Cell Culture

HaCaT was obtained from CLS (Cell Line Service, Eppelheim, Germany, ref. 3300493). HaCaT was cultured with DMEM (Sigma-Aldrich, Saint-Louis, MO, USA) supplemented with 10% heat-inactivated FBS (fetal bovine serum) (Sigma-Aldrich, Saint-Louis, MO, USA), 2% L-glutamine (Lonza, Basel, Switzerland), and 1% Penicillin-Streptomycin-Amphotericin B Mixture (Lonza, Basel, Switzerland). HaCaT cells were seeded at a density of 10 000 cells/cm^2^ and maintained at 37 °C in 5% CO_2_. The medium was changed twice a week. The cell confluence at the time of experiment was approximately 80%. 

### 2.5. Cell Treatment

Experimental group cells were treated with ME, ANDRO, NEO, 14DAP, or 14DAP11-12. The concentration range used was 1, 2.5 or 5 µg/mL for ANDRO, NEO, 14DAP, and 14DAP11-12, and the equivalent of 1, 2.5 or 5 µg/mL of andrographolide for ME. The control cells were treated with 0.05% DMSO (noted as “0”).

### 2.6. MTT Assay

Cell viability was assessed using a colorimetric assay that reduces MTT (3-(4,5-dimethylthiazol-2-yl)-2,5-diphenyltetrazolium bromide) (Sigma-Aldrich, Saint-Louis, MO, USA) to formazan dye, producing a purple color. Briefly, HaCaT was seeded in a 96-well plate at 14 × 10^3^ cells/well. After 24 h of incubation, cells were treated with the concentration range of ME, ANDRO, NEO, 14DAP, and 14DAP11-12 for 24 h or 48 h. Then, 10% (w/v) of MTT solution (5 mg/mL) was added to each well and further incubated for 4 h at 37 °C, 5% CO_2._ The medium was removed, and the blue crystals were dissolved in 100 µl SDS (Sodium dodecyl sulfate)-acidic-isopropanol solution (0.5% SDS; 80 mM HCl). The optical density (OD) of each well was measured at 450 nm using a 620 nm reference with a microplate reader (Multiskan GO Microplate Spectrophotometer, Thermo Scientific). The assay was performed in 6 replicates of three independent experiments (*n* = 3).

### 2.7. Lactate Dehydrogenase Activity 

Cell cytotoxicity was assessed by determining the amount of lactate dehydrogenase (LDH) released into the medium by damaged cells, using a Pierce LDH Cytotoxicity Assay Kit (Thermo Fisher Scientific Inc.). This method is based on the LDH-catalyzed reduction of pyruvate lactate by NADH. HaCaT was were seeded in 96-well plates at a density of 10 × 10^3^ cells per well. After 24 h of incubation, cells were treated with the concentration range of ME, ANDRO, NEO, 14DAP, and 14DAP11-12 for 24 h or 48 h. Briefly, equal amounts of culture supernatant were mixed with a reaction mixture containing NADH. After 30 min at room temperature, the reaction was stopped by Stop Solution. The absorbance was measured with a microplate reader (Multiskan GO Microplate Spectrophotometer, Thermo Scientific) at 490 nm using a 680 nm reference. LDH activity released during maximum LDH release by the complete lysis of cells were determined. Data are presented as the percentage of LDH released into the medium relative to maximum LDH control. The assay was performed in 6 replicates of three independent experiments (*n* = 3).

### 2.8. Intracellular Reactive Oxygen Species

The intercellular production of ROS levels was determined using 2′,7′-dichlorofluorescein diacetate (DCFH-DA; Sigma-Aldrich, Saint-Louis, MO, USA). The permeable DCFH-DA is oxidized by ROS to the highly fluorescent compound 2′,7′-chlorofluorescein (DCF). HaCaT was seeded in 96-well plates at 20 × 10^3^ cells/well. After 24 h of incubation, the medium was replaced by DMEM containing 25 µM DCFH-DA for 45 min at 37 °C. Then the DCFH-DA was removed, and the cells were washed with PBS (Dulbecco’s Phosphate Buffered Saline). Afterwards, the cells were incubated with the concentration range of ME, ANDRO, NEO, 14DAP, or 14DAP11-12 with or without 0.5 mM H_2_O_2_ (as free radical generator) for 1 h at 37 °C. Subsequently, fluorescence intensity per each well was detected using a microplate reader (EMax; Molecular Devices, Sunnyvale, CA) at an excitation wavelength of 485 nm and at an emission wavelength of 520 nm. Fluorescence intensity is directly proportional to the concentration of free radical compounds. The assay was performed in 6 replicates of three independent experiments (*n* = 3).

### 2.9. Quantitative RT-PCR

HaCaT was seeded in 6-well plates at 90 × 10^3^ cells/well up to 80% confluence. Then, the cells were pretreated with the concentration range of ME, ANDRO, NEO, 14DAP, or 14DAP11-12 for 18 h and Lipopolysaccharides (LPS; Sigma-Aldrich, Saint-Louis, MO, USA) was added to the medium at 10 µg/mL for an additional time of 6 h. Total RNA was isolated from cells using an RNeasy Mini Kit (Qiagen, Hilden, Germany) following the manufacturer’s instructions. Nucleic acid concentration and purity were determined by a μDrop™ plate (Thermo Fisher Scientific Inc.). One microgram of total RNA was retrotranscribed using a QuantiTect^®^ Reverse Transcription kit (Qiagen, Hilden, Germany) following the manufacturer’s procedure. The reaction was performed according to the manufacturer’s instructions for QuanTitect^®^ SYBR Green Master Mix (Qiagen, Hilden, Germany). Quantitative PCR (polymerase chain reaction) was performed by a C1000TM Thermal cycler (CFX96TM Real-Time System, Bio-Rad) under the following conditions: 10 min 95 °C, followed by 40 cycles of 15 s 95 °C and 1 min 60 °C. The quantitative PCR reaction was performed using specific primers: human TNF-α (Invitrogen: forward, 5′-CTC TTC TGC CTG CTG CAC TT-3′; reverse, 5′ CAG CTT GAG GGT TTG CTA CA3′) and GAPDH (Glyceraldehyde-3-phosphate dehydrogenase) (Qiagen cat. #QT00079247) as an internal control. Data were analyzed using the 2^−ΔΔCT^ method. The assay was performed in 2 replicates of three independent experiments (*n* = 3).

### 2.10. Measurement of IL-8 Secretion

HaCaT was seeded in 24-well plates at 19 × 10^3^ cells/well up to 80% confluence and then further cultured in fresh DMEM containing ME, ANDRO, NEO, 14DAP, or 14DAP11-12, with or without TNF-α (10 ng/mL, as cytokine generator) for 24 h. Supernatants were collected and used in the analysis of newly secreted interleukins. IL-8 was quantified using a sandwich ELISA assay kit (Peprotech, Rock Hill, NJ, USA), according to the manufacturer’s protocol. The assay was performed in 2 replicates of three independent experiments (*n* = 3).

### 2.11. Measurement of Hyaluronic Acid Secretion

HaCaT was seeded in 24-well plates at 19 × 10^3^ cells/well overnight and then further cultured in fresh serum-free DMEM with or without ME, ANDRO, NEO, 14DAP, or 14DAP11-12 for 48 h. Collected cell-free supernatants were analyzed for the level of HA by an ELISA kit (Echelon, Salt Lake City, USA) according to the manufacturer’s recommended protocol. The assay was performed in 2 replicates of three independent experiments (*n* = 3).

### 2.12. Statistical Tests

All data are presented as mean ± standard deviation (SD). Comparisons between groups were analyzed using GraphPad Prism software via ANOVA by the Kruskal–Wallis statistic (Dunn’s multiple comparisons test). Differences with *p*-value <0.05 were considered significant.

## 3. Results

### 3.1. Analysis of Methanolic Extract from Andrographis paniculata

**Figure 1:**
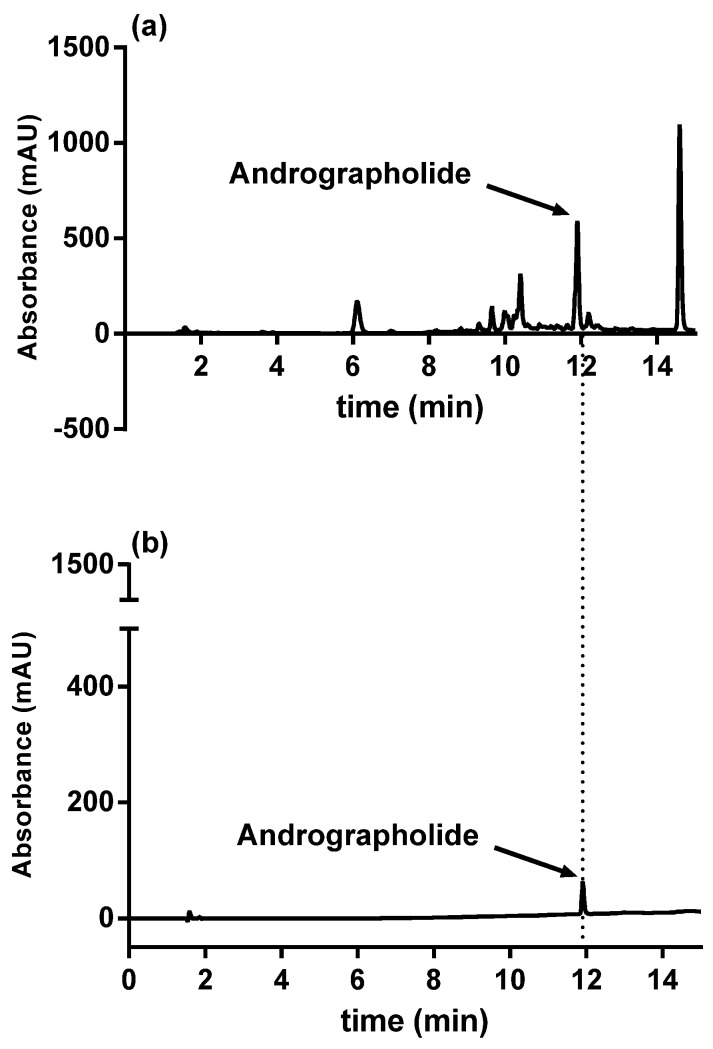
HPLC results of ANDRO from ME of *Andrographis paniculata* (**a**); ANDRO standard (**b**). Abbreviations: ANDRO, andrographolide; ME, methanolic extract.

ANDRO was identified in ME at 11.9 min by comparing the retention times obtained using a standard. We detected a proportion of ANDRO in ME of 0.87% (Figure 1). For the subsequent experiments, we tested ME according to its ANDRO concentration.

### 3.2. Cytotoxicity Assays

**Figure 2:**
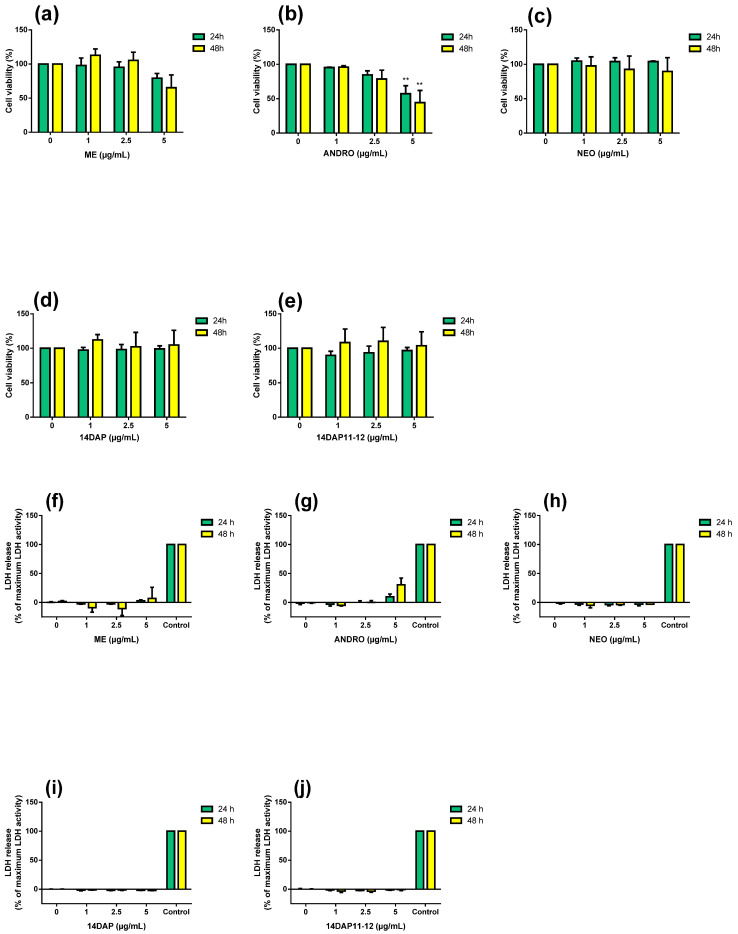
Effect of ME, ANDRO, NEO, 14DAP, and 14DAP11-12 on HaCaT cytotoxicity. HaCaT was treated with increasing concentrations (1, 2.5, or 5 µg/mL) of ME (**a**,**f**), ANDRO (**b**,**g**), NEO (**c**,**h**), 14DAP (**d**,**i**), or 14DAP11-12 (**e**,**j**) for 24 h and 48 h. The control cells were treated with 0.05% DMSO (noted as “0”). Cell viability was analyzed by mitochondrial metabolism using an MTT assay (**a**–**e**). Then, cell cytotoxicity was determined by a dosage of extracellular LDH activity (**f**–**j**). Cells untreated with stimuli were a negative control and cells treated with lysis agent were a positive control (noted as “Control”). The values are mean ± SD, ** *p* < 0.01 compared with control group, *n* = 3. Abbreviations: NEO, neoandrographolide; 14DAP, 14-deoxyandrographolide; 14DAP11-12, 14-deoxy-11,12-didehydroandrographolide; DMSO, dimethyl sulfoxide; MTT, 3-(4,5-dimethylthiazol-2-yl)-2,5-diphenyltetrazolium bromide; LDH, lactate dehydrogenase.

In the preliminary experiments, we evaluated the effect of ME, ANDRO, NEO, 14DAP, and 14DAP11-12 at several concentrations (1, 2.5, and 5 µg/mL) in HaCaT for 24 h and 48 h. Specifically, cell viability was analyzed by mitochondrial activity using an MTT assay (Figure 2a–e). Moreover, cell cytotoxicity was assayed using a dosage of extracellular LDH activity (Figure 2f–j). Only ANDRO significantly decreased cell viability at 5 µg/mL at 24h and 48h (Figure 2b). Our treatments did not increase cytotoxicity (Figure 2f–j). Therefore, ME, NEO, 14DAP, and 14DAP11-12 were used in subsequent experiments at 1, 2.5, and 5 µg/mL, except for ANDRO, which was used at 1 and 2.5 µg/mL. 

### 3.3. Antioxidant Activity 

**Figure 3:**
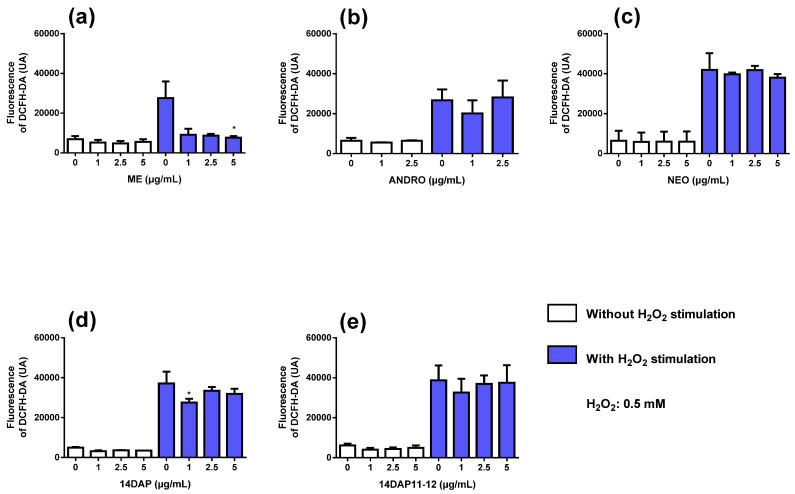
ROS production and the effect of ME, ANDRO, NEO, 14DAP, and 14DAP11-12 in HaCaT. ME (**a**), ANDRO (**b**), NEO (**c**), 14DAP (**d**), or 14DAP11-12 (**e**) was used at 1, 2.5, or 5 µg/mL for 1 h. The control cells were treated with 0.05% DMSO (noted as “0”). ROS production was induced by 0.5 mM H_2_O_2_ and free radical scavenging activity was done using a DCFH-DA probe. The values are mean ± SD, * *p* < 0.05 compared with control group, *n* = 3. Abbreviations: ROS, reactive oxygen species; DCFH-DA, 2′,7′-dichlorofluorescein diacetate.

Next, we analyzed the effect of ME, ANDRO, NEO, 14DAP, and 14DAP11-12 on ROS production in HaCaT for 1h. Co-treatment with H_2_O_2_ caused an increase of intracellular ROS compared to unstimulated cells (Figure 3). ME at 5 µg/mL (Figure 3a) and 14DAP at 1 µg/mL (Figure 3d) significantly reduced ROS production in H_2_O_2_-stimulated HaCaT compared to control cells treated with H_2_O_2_ (72% and 14% decreases, respectively). 

### 3.4. Anti-Inflammatory Activities

**Figure 4:**
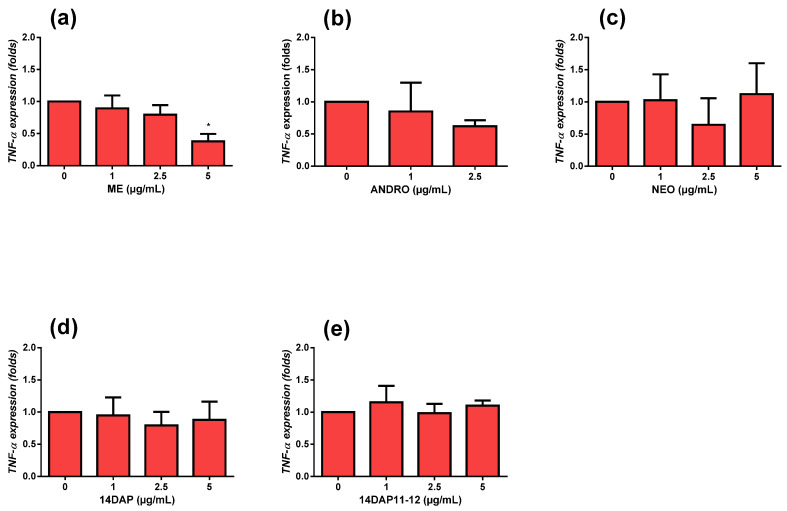
TNF-α expression and effect of ME, ANDRO, NEO, 14DAP, and 14DAP11-12 in HaCaT under pro-inflammation conditions. ME (**a**), ANDRO (**b**), NEO (**c**), 14DAP (**d**), or 14DAP11-12 (**e**) was used at 1, 2.5, or 5 µg/mL for 24 h. The control cells were treated with 0.05% DMSO (noted as “0”). Inflammation conditions were induced by LPS (10 µg/mL) for 6 h and TNF-α expression was determined by RT-qPCR. The values are mean ± SD, * *p* < 0.05 compared with control group, *n* = 3. Abbreviations: LPS, lipopolysaccharides; RT-qPCR, reverse transcription-quantitative polymerase chain reaction; TNF-α, tumor necrosis factor-α.

To explore TNF-α expression, we pretreated HaCaT with ME, ANDRO, NEO, 14DAP, or 14DAP11-12 for 18 h. Then, we co-stimulated the cells with LPS at 10 µg/mL for an additional 6 h. Under pro-inflammatory conditions, ME at 5µg/mL decreased TNF-α expression significantly (Figure 4a).

**Figure 5:**
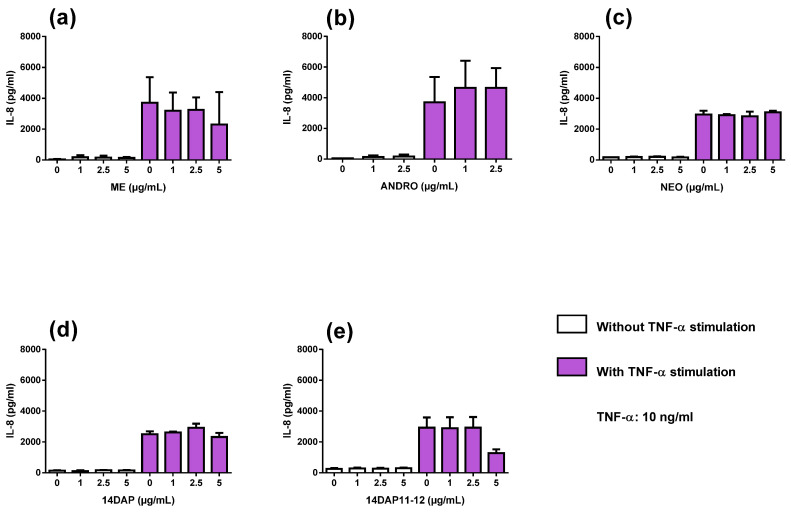
IL-8 secretion and effect of ME, ANDRO, NEO, 14DAP, and 14DAP11-12 in HaCaT under pro-inflammation conditions. ME (**a**), ANDRO (**b**), NEO (**c**), 14DAP (**d**), or 14DAP11-12 (**e**) was used at 1, 2.5, or 5 µg/mL for 24 h. The control cells were treated with 0.05% DMSO (noted as “0”). Inflammation was induced by TNF-α (10 ng/mL) and IL-8 secretion was performed using an ELISA assay. The values are mean ± SD, compared with control group, *n* = 3. Abbreviations: IL-8, interleukin-8.

We observed an increase of IL-8 secretion in TNF-α-stimulated HaCaT compared to unstimulated cells (Figure 5). However, IL-8 secretion was not reduced by our treatments in HaCaT under TNF-α stimulation (Figure 5).

### 3.5. Hyaluronic Acid Production

**Figure 6:**
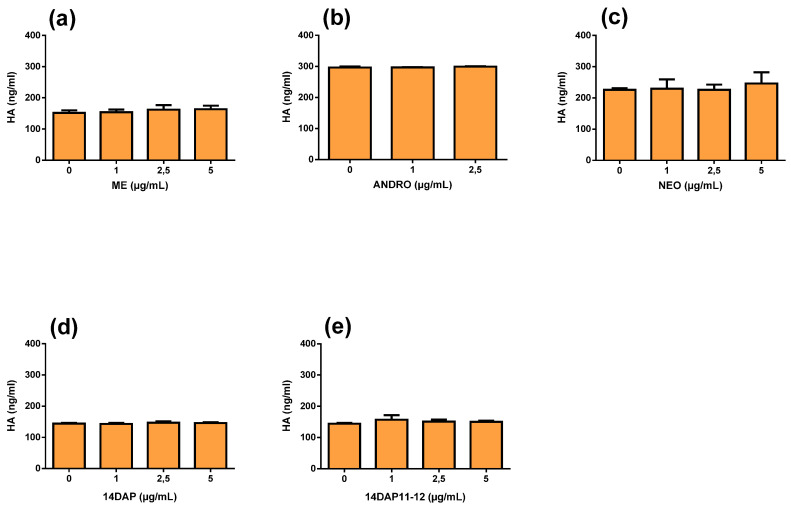
HA production and effect of ME, ANDRO, NEO, 14DAP, and 14DAP11-12 in HaCaT. ME (**a**), ANDRO (**b**), NEO (**c**) 14DAP (**d**), or 14DAP11-12 (**e**) was used at 1, 2.5 or 5 µg/mL for 48h. HA was determined by an ELISA. The values are mean ± SD, compared with control group, *n* = 3. Abbreviations: HA, hyaluronic acid.

Finally, we measured HA synthesis in HaCaT treated with ME, ANDRO, NEO, 14DAP, or 14DAP11-12 for 48 h (Figure 6). As shown in Figure 6, there was no effect on HA production for all treatments.

## 4. Discussion

*Andrographis paniculata* is a traditional plant used in Asia to treat various diseases such as intestinal disorders, influenza epidemics, and other respiratory infections [24,25]. ANDRO is a major bioactive compound isolated from this plant [26]. For example, the anti-inflammatory activity of *Andrographis paniculata* is commonly attributed to ANDRO and its analogs [10]. A synthetic analog of ANDRO prevented UVB (Ultraviolet B)-induced premature aging in mouse skin [27]. In this study, we evaluated the anti-aging properties of ME, ANDRO, NEO, 14DAP, and 14DAP11-12 on human keratinocytes. 

In the present study, ME contained 0.87% ANDRO (Figure 1). Other studies have identified over 20 diterpenes and over ten flavonoids in the same type of extract [28]. ANDRO has been isolated and identified up to 4% in whole plants, between 0.8% and 1.2% in stems, and between 0.5% and 6% in dried leaves [26,28,29]. Our extract was obtained from the leaves; its ANDRO level was low, but ranging between 0.5% and 6%.

In our experiments, ME (5 µg/mL) and 14DAP (1 µg/mL) showed an antioxidant effect in keratinocytes (Figure 3). Antioxidant compounds are given special attention to prevent skin damage. *Andrographis paniculata* extracts are a source of powerful antioxidants [13,30]. The solvent of extraction plays an important role in the composition of antioxidant compounds. For example, the inhibition of lipid peroxidation and free radical scavenging activity is more effective in methanolic extraction than in aqueous extraction [31]. In methanolic extract, a higher concentration of ANDRO and 14DAP11-12 was found [31]. In a primary culture of human dermal fibroblasts (HDFa), ME (5 µg/mL) and 14DAP (1 µg/mL) decreased ROS production under oxidative stress conditions [14]. Andrographolide sodium bisulfate is a soluble product derived from ANDRO. In HaCaT, pretreatment with andrographolide sodium bisulfate reduced excessive UV-induced ROS levels using the Keap1/Nrf2 pathway [32]. ANDRO is involved in regulation of the antioxidant defense system by the Keap1/Nrf2 pathway [16]. DCFH-DA is the most widely used probe to detect H_2_O_2_-induced oxidative stress. However, there are limitations associated with the DCFH-DA test. For example, we could measure intracellular and mitochondrial superoxide using hydroethidine and Mito-SOX^TM^ (Red Mitochondrial Superoxide Indicator kit).

Under inflammatory conditions, ME (5 µg/mL) significantly reduced TNF-α expression (Figure 4), but not IL-8 secretion (Figure 5) in HaCaT. Our results regarding TNF-α mRNA support several previous studies, including studies of the human monocyte cell line THP-1 [33]. In HDFa cells, we showed that ME (5 µg/mL) and ANDRO (5 µg/mL) treatments decreased TNF-α expression under inflammatory conditions [14]. Compounds from *Andrographis paniculata* also regulated TNF-α protein. For example, andrographolide sodium bisulfate reduced excess levels of TNF-α in mice skin associated with UV exposure [27]. In HaCaT, andrographolide sodium bisulfate downregulated the protein expression of p65 and decreased the production of TNF-α [32]. Regarding IL-8 secretion, it has been reported that ANDRO inhibits TNF-α-stimulated IL-8 expression in HCT116 cells by suppressing NADPH oxidase activation and ROS generation [34]. In HDFa cells, ME, ANDRO, NEO, 14DAP, and 14DAP11-12 were found not to reduce IL-8 secretion [14]. 

ME, ANDRO, NEO, 14DAP, and 14DAP11-12 did not change HA secretion (Figure 6). In a primary cartilage culture treated with IL-1b, ANDRO reduced HA secretion [34]. In UV-induced skin photoaging of mice, andrographolide sodium bisulfate increased water content [27].

## 5. Conclusions

In this study, we demonstrated the beneficial effect of ME against oxidative stress and inflammation in keratinocytes. Herein, ME decreased ROS production and TNF-α expression in HaCaT under pro-oxidant and pro-inflammatory conditions, respectively.

## Figures and Tables

**Table 1:** Structure of ANDRO, NEO, 14DAP and 14DAP11-12.

Name.	Structure and Details
Andrographolide (ANDRO)	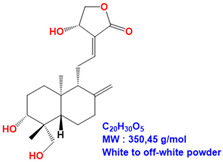
Neoandrographolide (NEO)	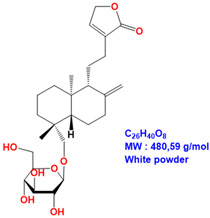
14-deoxyandrographolide (14DAP)	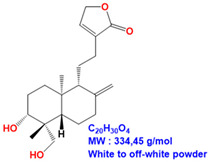
14-deoxy-11,12-didehydroandrographolide (14DAP11-12)	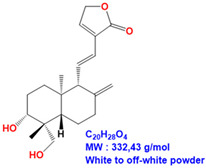

## Abbreviations

14DAP	14-deoxyandrographolide
14DAP11-12	14-deoxy-11,12-didehydroandrographolide
ANDRO	andrographolide
ECM	extracellular matrix
HA	hyaluronic acid
HDFa	human dermal fibroblasts, adult
IL-8	interleukin-8
LDH	lactate dehydrogenase
ME	methanolic extract
MMPs	matrix metalloproteinases
NEO	neoandrographolide
ROS	reactive oxygen species
TNF-α	tumor necrosis factor-α
